# Metal Mesh and Narrow Band Gap Mn_0.5_Cd_0.5_S Photocatalyst Cooperation for Efficient Hydrogen Production

**DOI:** 10.3390/ma15175861

**Published:** 2022-08-25

**Authors:** Haifeng Zhu, Renjie Ding, Xinle Dou, Jiashun Zhou, Huihua Luo, Lijie Duan, Yaping Zhang, Lianqing Yu

**Affiliations:** 1 School of Science, China University of Petroleum, Qingdao 266580, China; 2 College of Materials Science and Engineering, China University of Petroleum, Qingdao 266580, China

**Keywords:** co-catalyst system, Mn_0.5_Cd_0.5_S, metal and alloy mesh, H_2_ evolution, photoinduced electron transfer

## Abstract

A novel co-catalyst system under visible-light irradiation was constructed using high-purity metal and alloy mesh and a Mn_0.5_Cd_0.5_S photocatalyst with a narrow band gap (1.91 eV) prepared by hydrothermal synthesis. The hydrogen production rate of Mn_0.5_Cd_0.5_S changed from 2.21 to 6.63 mmol·(g·h)^−1^ with the amount of thioacetamide, which was used as the sulphur source. The introduction of Ag, Mo, Ni, Cu, and Cu–Ni alloy meshes efficiently improved the H_2_ production rate of the co-catalyst system, especially for the Ni mesh. The improvement can reach an approximately six times greater production, with the highest H_2_ production rate being 37.65 mmol·(g·h)^−1^. The results showed that some bulk non-noble metal meshes can act as good or better than some noble metal nanoparticles deposited on the main photocatalyst for H_2_ evolution due to the promotion of photoinduced electron transfer, increase in redox reaction sites, and prevention of the recombination of carriers.

## 1. Introduction

The production of hydrogen through the solar photolysis of water is one potential route with great advantages to solve the current global energy and environmental crisis. From previous work that was focused solely on the pursuit of high-efficiency hydrogen production, the field has gradually developed to a stage in which other factors of the photocatalyst should be considered, such as low cost, high stability, simple preparation, and even effective practical application [1,2,3,4]. Generally, improving photocatalytic efficiency can be feasibly achieved by synthesizing different composite catalysts for increasing light harvest. By introducing co-catalysts to modify and form type II or Z-scheme construction, which can promote the separation of carriers and increase redox reaction sites, the catalytic efficiency of the catalyst system can be greatly improved [1]. In 1998, Fujihara et al. [5] combined the electrochemical system to construct a Z-type heterojunction photocatalytic system, which not only required the Nafion membrane as a proton transport membrane to separate the anode chamber and cathode chamber of the photoreaction system, but also utilizes Br^−^/Br_2_ and Fe^3+^/Fe^2+^ as redox pairs. As such, the disadvantages of adopting co-catalysts and forming heterojunctions are quite obvious; for example, there is shielding and competition between the co-catalysts and main catalyst, so oxidation–reduction pairs are required to attenuate this effect, leading to high costs and harsh service conditions [1]. Many subsequent studies, such as Wang et al. [6], have deposited the precious metals Au and Rh on the surfaces of TiO_2_ and SrTiO_3_, respectively and then combined them to form a Z-scheme transmission structure. Although this modified treatment successfully removed the redox pair, there are still unfavourable aspects such as high cost and complex processes in practical industrial applications.

In 1982, Pichat et al. [7] deposited the precious metal Pt on the surface of TiO_2_ in the form of atomic clusters and realized the improvement of H_2_ production in the photocatalytic system with fatty alcohol as a sacrificial reagent. The Pt used in the study has the largest work function W in precious metals, which is calculated by the following Formula (1):

(1) $${W=\chi -E}_{F}$$

As in the aforementioned studies, the co-catalytic system constructed with precious metal could effectively improve hydrogen production, but practical application was limited by the high cost. Compared to the noble metals, some transition metals with a low price and relatively good conductivity can also demonstrate good performance. Furthermore, the work function can be adjusted by forming alloys with different ratios of transition metals to meet the requirements of hydrogen production. However, the method of improving photocatalytic performance by contacting non-nanoparticle metal seems to have been neglected, which might be simpler and more convenient.

As a metal sulphide and visible light-responsive semiconductor with an adjustable energy band structure, CdS has been widely studied in the field of photocatalysis because of its narrow band gap and high hydrogen production rate [8,9,10,11,12,13,14,15,16]. However, it is difficult to apply on a large scale due to its serious photo-corrosion and rapid recombination of electron–hole pairs [17,18]. Therefore, to improve the photocatalytic activity and stability of photocatalysts based on CdS, people have used methods using composites with nanomaterial materials to construct heterojunctions [12,13,14], modify the co-catalyst [14,15,16], and prepare multicomponent solid solutions [19,20,21,22,23]. Multi-component solid solutions in which the band gap can be adjusted by changing the composition ratio usually exhibit better activity than cadmium sulphide alone, and the potential requirements of H^+^/H_2_ and O_2_/H_2_O in hydrogen production can be met more easily. In 2010, Ikeue et al. [24] first reported the Mn_1−x_Cd_x_S solid solution, which not only has good visible light-driven H_2_ production activity but also possesses excellent photo-corrosion resistance. In order to further improve the activity and stability of the Mn_1−x_Cd_x_S photocatalyst, the precious metal Pt has been loaded on Mn_1−x_Cd_x_S in some works.

Herein, a novel Mn_0.5_Cd_0.5_S material was prepared by hydrothermal synthesis and used as a light absorber. A simple co-catalyst system was formed with Mn_0.5_Cd_0.5_S and a metal mesh directly placed into the sacrificial agent. Adopting different metal meshes, the photocatalytic performance of the co-catalyst system can be greatly improved. Meanwhile, a possible mechanism for the enhanced photocatalytic activity of Mn_0.5_Cd_0.5_S/metal mesh was proposed in combination with various characterizations. Different from the recombination of metal nanoparticles with a main catalyst, this concerted catalysis using a direct physical contact pattern may yield more merits for practical application, such as simple preparation of catalyst, low cost, weak impairment of the environment, etc.

## 2. Experimental

### 2.1. Chemical Reagents

Sodium hydroxide (NaOH), manganese acetate tetrahydrate (Mn(Ac)_2_·4H_2_O), cadmium acetate dihydrate (Cd(Ac)_2_·2H_2_O), and thioacetamide (TAA) were all from obtained from Aladdin (Shanghai, China) and are analytically pure, i.e., they can be used without further purification.

### 2.2. Preparation of Mn_0.5_Cd_0.5_S Material

The Mn_0.5_Cd_0.5_S products (hereafter referred to as MCS) were synthesized through the one-pot solvothermal process. The synthesis method was an improved method based on previous studies [25]. A total of 1 mmol Mn(Ac)_2_·4H_2_O and 1 mmol Cd(Ac)_2_·2H_2_O were dissolved in 40 mL deionized water with stirring for 10 min and designated as solution A. Additionally, 6 mmol thioacetamide (TAA) was added into 40 mL deionized water while the pH of the solution was adjusted to 10.5 using NaOH (6 mol/L) and designated as solution B. Solution A was then added dropwise to solution B at a constant flow rate. The mixture, which had been stirred for 30 min, was added to a Teflon-lined autoclave (100 mL), then heated to 130 °C and kept for 10 h. The products were washed with deionized water and absolute ethyl alcohol three times each and the final samples were obtained by drying at 60 °C. A series of derivatives was synthesized depending on the amount of TAA added during the synthesis process, and the names of the various products can be found in Table 1.

### 2.3. Preparation of Metal Mesh

Metal meshes of Ag, Mo, Ni, Cu and Cu–Ni alloy (Tengyun, Xingtai, China) were selected as the main objects in this work. Preference was given to metal with less impurity. Among them, nickel metal with grade N6 (UNS NO2200) was selected as the raw material of high-purity metal nickel mesh, in which the content of Ni was greater than 99.5% and the content of other impurities was not higher than 0.5%. In addition, Monel alloy (UNS NO4400, nickel content 63.0–70.0%, copper content 28.0–34.0%) was selected as the raw material for the copper–nickel alloy mesh. Each metal mesh was processed into a circle with a radius of 4 cm and was consistent with the irradiation area of the reactor. The mesh number of every metal mesh was 40, and the surface was polished with sandpaper before addition to the reaction system.

### 2.4. Material Characterization

The PANalytical XPert PRO MRD system (XRD) with Cu-Kα radiation (λ = 1.5418 Å) was mainly used in the crystal phase analysis (40 kV, 30 mA, and scan rate 0.07° s^−1^ in the range of 2θ = 20–80°). The micromorphology of the samples was surveyed using a TM4000Plu electron microscope, and the component content of the metal mesh was obtained by matching EDX components. A T9+ PERSEE UV-vis spectrophotometer with 150 mm integrating sphere was used to measure the UV-vis spectra of the synthetic samples using a test range of 200–800 nm. A JME-2100 transmission electron microscope was used to obtain the lattice diffraction fringes of materials and analyse the phase composition. A Thermo Scientific EACALAB Xi+ photoelectron spectrometer (XPS) with monochromatic AL-KA as the radiation source was used to analyse the constituent elements and their chemical environment. The vacuum degree was approximately 2 × 10^−9^ mbar, the energy of the X-ray source was 1486.6 ev, the voltage was 15 kV, the beam current was 10 mA, and the analyser scans were conducted in CAE mode.

The photoelectrochemical measurements were performed on a CHI660E electrochemical workstation (Chenhua, Shanghai, China) using a three-electrode system. The catalyst-coated FTO glass (1 × 2 cm^2^) was used as a work electrode, the Ag/AgCl was used as a reference electrode (3.5 M/KCL, 0.205 V vs. NHE), and a 52-mesh pure platinum mesh was used as a counter electrode (10 mm × 10 mm). Na_2_SO_4_ (0.5 M) aqueous solution was used as the electrolyte. The photocurrent (I-t) test was started in a shaded state after 2 s at rest, the light was turned on after 50 s, maintained for 50 s, and then the test returned to a shaded state again, alternating cycles of 400 s duration. Electrochemical impedance spectroscopy (EIS) was tested with a biasing voltage of 0.2 V s^−1^. The Mott–Schottky value was obtained at a frequency of 1000 Hz and then the E_fb_ value was determined from the Mott–Schottky relationship.

### 2.5. Photocatalytic Performance Tests

**Figure 1:**
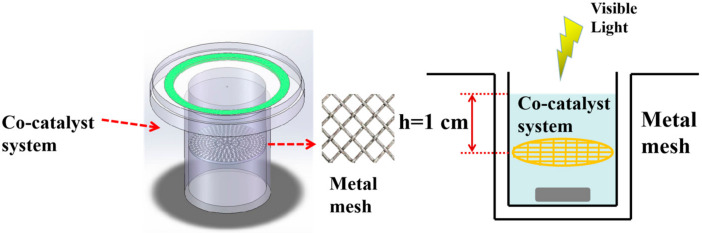
Schematic diagram of the reactor loaded with metal mesh.

The entire process of the photocatalytic hydrogen production performance tests was carried out in a sealed-top irradiated reaction vessel. During the whole reaction, the temperature was controlled at 6 °C using a circulating condensation device. The lamp source was 10 cm away from the solution surface, and the stirring speed of a magnetic stirrer was set to 300 rpm. A total of 5 mg of photocatalyst and polished metal mesh was added as needed, and finally, uniformly dispersed by ultrasound for 10 min. A 300 W Xe-lamp was selected to simulate the solar light source, the current was set to 15 mA, and a 420 nm cutoff lens (λ ≥ 420 nm) was used to filter out the UV light. Accurate H_2_ production performance was tested mainly with a web-linked gas chromatograph detection system (CEAULIGHT, 5 Å reference column, TDX-01 detector, and high-purity N_2_ (≥99.999%) was used as the carrier gas). In addition, it should be noted that the air in the reaction system should be completely removed before irradiation. Figure 1 shows the reactor section of experimental setup for the H_2_ production. The metal mesh was placed 1 cm below the liquid surface so that both the mesh and the catalysts in the system were exposed to light radiation.

The appropriate band-pass filters were selected to be loaded on the 300 W Xe-lamp to obtain the desired monochromatic light for the apparent quantum yield (AQY) measurements. Meanwhile the CEL-NP2000 Optical Power Meter (CEAULIGHT, CHN, accuracy of 0.001 mw/cm^2^, Beijing China Education Au-light Technology Co., Ltd., Beijing, China) was used to test the light intensity. AQY values were calculated according to the following Equation (2):

(2) $$\mathrm{AQY}(\%)=\frac{2\times \mathrm{number}\;\mathrm{of}\;H_{2}\;\mathrm{molecules}}{\mathrm{number}\;\mathrm{of}\;\mathrm{incident}\;\mathrm{photons}}\times 100$$

## 3. Results and Discussion

### 3.1. Crystal Phase and Composition Analyses

**Figure 2:**
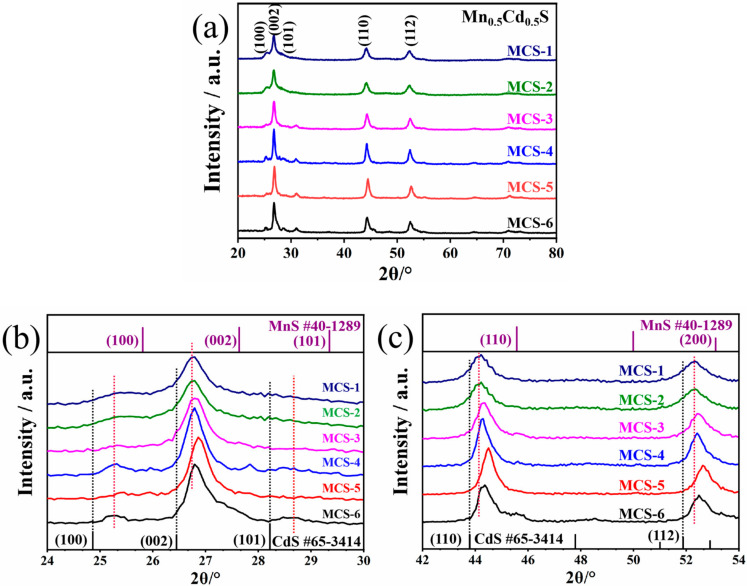
(**a**) XRD patterns of the MCS products with different TAA addition. (**b**) XRD diffraction patterns of 24–30°. (**c**) XRD diffraction patterns of 42–54°.

Figure 2 depicts the XRD patterns of various products obtained by solvent thermal treatment at 130 °C. As can be observed in Figure 2a, the peaks of Mn_0.5_Cd_0.5_S products with different amounts of TAA addition are in agreement with previously reported values [26], exhibiting characteristic peaks at 2θ = 25.0°, 26.7°, 28.4°, 44.0° and 52.33°, which correspond to the (100), (002), (101), (110), and (112) facets of the MCS solid solution, respectively.

In order to further prove the successful preparation of the MCS solid, Figure 2b,c introduces the standard diffraction peaks of hexagonal wurtzite γ-MnS (JCPDS Card No. 40-1289) and hexagonal CdS (JCPDS Card No. 65-3414). It can be seen that the diffraction peaks with less intensity are located at 2θ = 27.6° and 45.6°, which can be ascribed to the reflection of the (002) and (110) planes of γ-MnS. Related studies have shown that the metastable γ-MnS crystal state can be synthesized by solvothermal reaction at 190–200 °C [27]. Therefore, although unstable phases such as β-MnS and γ-MnS can be easily converted to α-MnS under high-temperature or high-pressure conditions, the appearance of γ-MnS in this work’s hydrothermal synthesis condition is still explicable [4,24,28].

In addition, compared to the (100), (002), (101), (110), and (112) diffraction peaks of hexagonal CdS, MCS products slightly shifted toward higher diffraction angles [29]. The changes in the diffraction peaks demonstrate that these products were not a simply MnS/CdS mixture but Mn_0.5_Cd_0.5_S solid solution. Due to the fact that the ionic radius of Mn^2+^ (0.46 Å) is smaller than that of Cd^2+^ (0.97 Å), Mn^2+^ can be brought into the CdS lattice or its interstitial sites [4,29]. It is worth noting that when the positions of the diffraction peaks of the MCS-1 sample were marked by red dashed lines in Figure 2b,c, it is clearly visible that with the increase in TAA addition, the offset to high angle of each sample increases and then decreases, reaching the maximum offset at the MCS-5 sample. Combined with the above analyses, it was determined that the dissolution of γ-MnS in CdS crystals varied with the addition of TAA during the present hot solvent synthesis. However, this variation is not a simple positive linear relationship, as the increase in the amount of TAA in a certain range can promote the formation of an MCS solid solution. This positive facilitation may be attributed to the fact that the CdS lattices or interstitial sites are more exposed to the surface with increasing amounts of TAA, providing more solubilization sites for Mn^2+^. When the TAA addition is large enough, (such as in MCS-6, where the TAA content is 20 times that of Mn^2+^ and Cd^2+^ in the system), contact between MnS and CdS is made difficult, thus weakening the promotion effect brought by the exposed lattice or interstitial sites.

### 3.2. Microstructure Analyses

**Figure 3:**
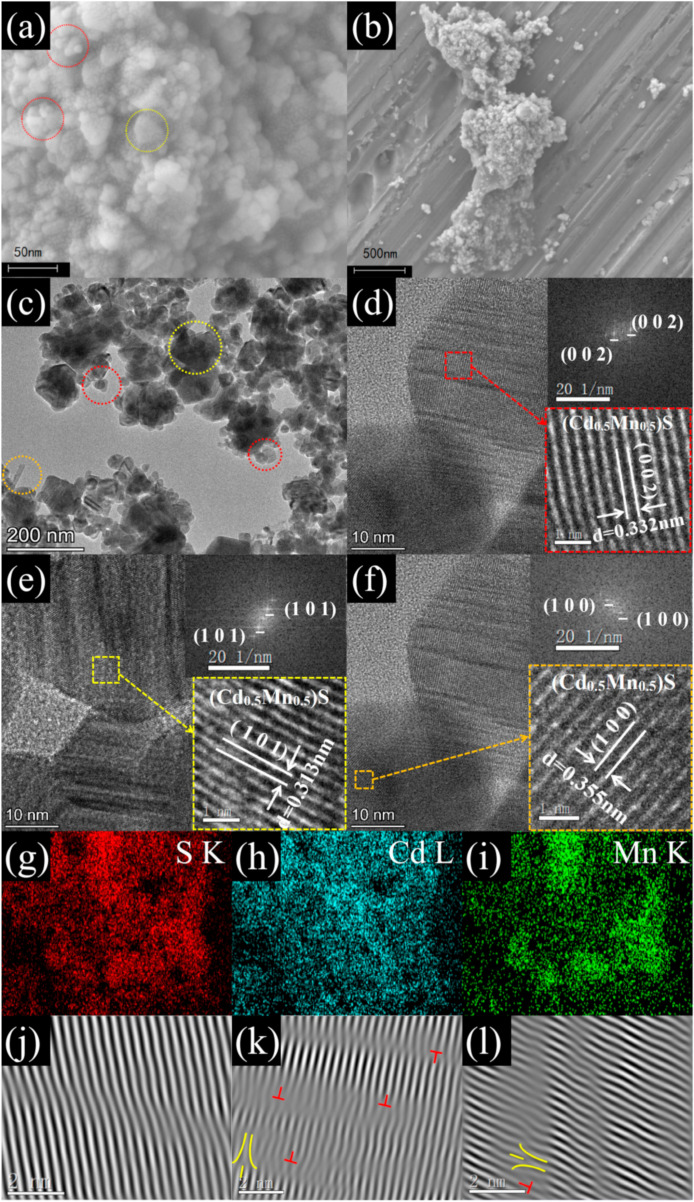
(**a**,**b**) SEM images of MCS-2 samples. (**c**–**f**) HRTEM image of the MCS-2. (**g**–**i**) EDX element mappings of MCS-2. (**j**–**l**) Inverse fast Fourier transform patterns of the (002), (101), and (100) planes of MCS-2.

The microscopic appearance of the MCS-2 is shown in Figure 3a,b. It can be clearly observed that the MCS-2 crystals have two morphologies, one which is a hexagonal nanometre lamellar morphology with a width of about 40 nm (as shown in the yellow circle in Figure 3a), and the other is a microsphere-like structure with a width about 20 nm (as shown in the red circle in Figure 3a).

In Figure 3c, it can be seen that the sample mainly consisted of hexagonal nanometre lamellar and nanospheres with lengths ranging from 20 to 200 nm. In addition, a small amount of rod-like morphologic structure can be observed, with a width of about 150 nm. From the HRTEM image (Figure 3d), it can be seen that the lattice spacing (0.332 nm) of the MCS-2 lattice stripe (shown in red square in Figure 3d) is a little lower than the lattice spacing (0.336 nm) of the hexagonal nanometre lamellar CdS (002) plane. Similarly, the other lattice spacing (0.313 nm and 0.355 nm, as shown in the yellow and brown square in Figure 3e,f) is also slightly lower than lattice spacing (0.316 nm and 0.358 nm) of the (101) and (100) plane of hexagonal CdS [4]. Combined with the EDX elemental mapping results in Figure 3g–i, the uniform distribution of S, Cd, and Mn indicates that the reduced lattice spacing of cadmium sulphide is due to the doping of Mn^2+^ ions in its lattice under the current solvent heat conditions and provides strong evidence for the successful preparation of the Cd–Mn–S ternary phase. In addition, the similar hexagonal crystal phase of MCS-2 and CdS can be demonstrated using the fast Fourier transform (FFT) pattern (inset in the right upper corner of Figure 3d–f).

Figure 3g–i are the dislocation characterization results from the (002), (100), and (101) planes of MCS, respectively. Obvious screw dislocations (marked with red marks) and edge dislocations (marked with yellow marks) can be seen in Figure 3h,i. The dislocation density is relatively high in Figure 3h,i, while there is no obvious dislocation phenomenon in Figure 3g. In the process of MnS and CdS forming a solid solution, the integration of solute atom Mn will inevitably cause lattice distortion. Additionally, the increase in shear stress due to doping provides the impetus for the occurrence of dislocation motion, which causes slip. Therefore, it can be determined that the growth of the MCS solid solution is most difficult on the (002) crystal plane, which has the lowest dislocation density. Its higher relative peak intensity with respect to the (100) and (101) crystal planes is primarily due to the good crystallization of hexagonal CdS on this crystal plane. Therefore, it can be determined that the preferred growth direction of the MCS solid solution is along the (100) and (101) crystal planes [2].

**Figure 4:**
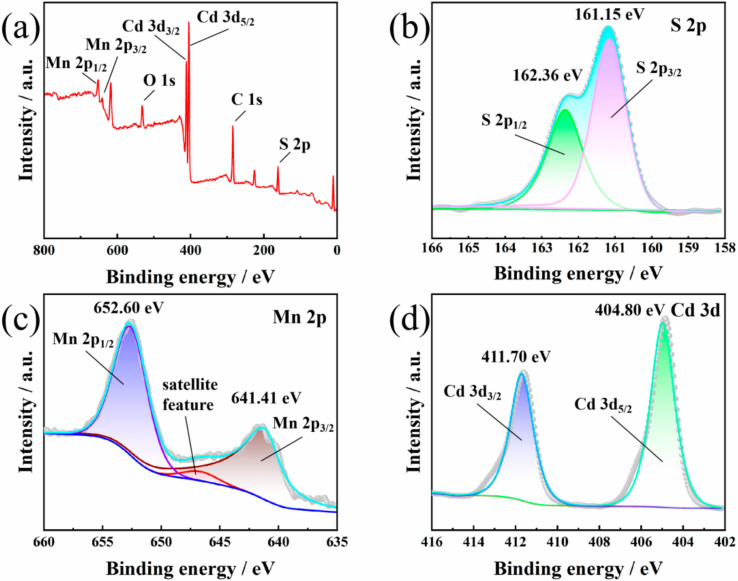
(**a**) XPS survey spectra of MCS-2. (**b**–**d**) High-resolution XPS spectra of (**b**) S 2p, (**c**) Mn 2p, and (**d**) Cd 3d in MCS-2 composite.

The composition and state of the surface elements of MCS-2 were obtained with X-ray photoelectron spectroscopy (XPS). The Mn, Cd, and S elements were observed in survey spectra in Figure 4a; this provides more evidence of the successful preparation of the MCS composite [4,25,30]. A spin-orbital splitting peak can be seen in Figure 4b, which appears due to the fact that S 2p has a dense spin-orbit component (Δ = 1.16 eV) and the splitting of the subshell layer on its p orbital. Additionally, two apparent symmetrical peaks located at 161.15 and 162.36 eV are ascribed to S 2p_3/2_ and S 2p_1/2_, indicating that the S exists in a −2 oxidation state [25,31]. Multiple splitting peaks are visible in the high-resolution Mn 2p XPS spectrum in Figure 4c: the major peaks appear at 641.41 and 652.60 eV, which can be attributed to Mn 2p_3/2_ and Mn 2p_1/2_, respectively. At the same time, the satellite peak appearing at 645.98 eV reveals a +2 oxidation state of Mn in the MCS-2 composite [3,30]. As shown in Figure 4d, the peaks that appear at 404.80 and 411.70 eV are ascribed to Cd 3d_5/2_ and Cd 3d_3/2_, respectively, indicating that the Cd in MCS-2 presents in the +2 oxidation state [30]. The above binding energies of Cd, Mn, and S are in good agreement with the reported values.

**Figure 5:**
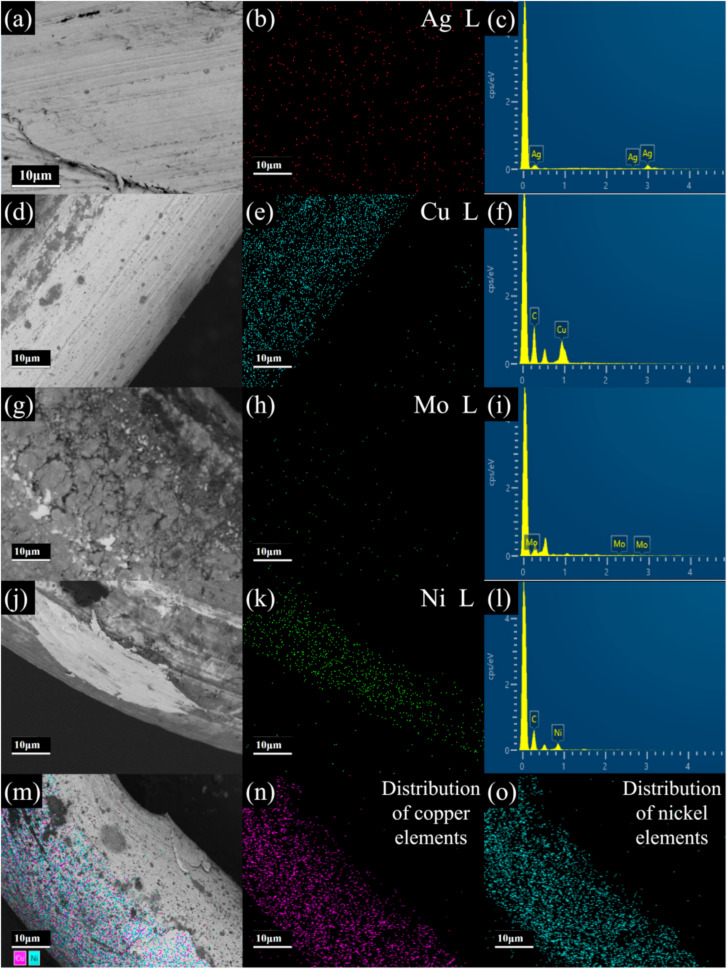
(**a**–**o**) Different metal mesh SEM image, mapping, and EDX spectra.

The energy dispersive X-ray element mappings (Figure 4a–e) indicate that the metal mesh used in the catalytic process is highly pure. In all of them, with the exception of the high-purity copper mesh and high-purity nickel mesh group, the spectral peak of C appears in the K-line system due to the participation of carbon (C)-containing conductive adhesive in the scanning during the test, which are 100% in atomic proportion. The mapping spectrum of the copper nickel alloy in Figure 5e shows that its composition distribution is homogeneous, while its actual composition content can be calculated by EDX, as shown in Table 2.

### 3.3. Absorption Spectra Analyses

**Figure 6:**
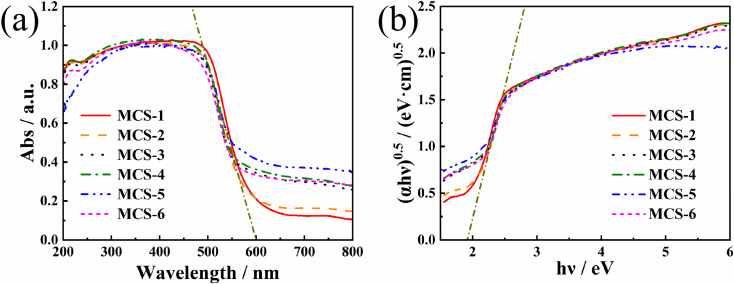
(**a**) UV-vis diffuse reflectance spectra of the MCS samples with a absorption edge located around 600 nm. (**b**) (αhʋ)^0.5^ versus hʋ plots of the MCS samples.

The light-harvesting ability of the catalyst was evaluated through UV-vis diffuse reflectance spectrometry. Figure 6 shows the UV-vis diffuse reflectance absorption spectra and Tauc curves of MCS products, respectively. In drawing the Tauc curves, we finally determined the band gap type of MCS to be an indirect band gap by reviewing the relevant literature [4,26,32] in combination with Figure 6a to make a rough judgment. As can be seen in Figure 6a, all MCS samples have absorption in the 200–800 nm wavelength. Meanwhile, all the MCS samples have a close absorption edge located around 600 nm, corresponding to a bandgap energy of approximately 2 eV. Additionally, the absorption intensity is lower in the range of 600–800 nm, and the mean absorbance is listed in Table 3.

In Table 3, the background absorptions of MCS composites in the range of 600–800 nm exhibited trend that first increased and then decreased along with the increasing TAA content. As can be seen in Figure 6a, MCS-5 has the strongest absorption capacity in the 600–800 nm range. Owing to the increase in light absorption, more electrons can be generated to participate in H_2_ production reactions [4], and therefore MCS-5 can be predicted to have excellent hydrogen production performance. Moreover, the accurate band gap (E_g_) of the MCS-2 was determined as [33,34].

(3) $${\left(\alpha hʋ\right)}^{0.5}=B\left(hʋ-\mathrm{Eg}\right)$$

where α is absorption coefficient, E_g_ is the band gap energy, *B* is a constant, and hʋ is the photon energy.

In Figure 6b, the band gap of MCS-2 was finally determined to be 1.91 eV, which is in the excitement range of visible light. As previously mentioned, the one-pot solvothermal process MCS-2 has a small band gap (1.91 eV), which is smaller than the reported (2.23 eV) [25], and it is more favourable for photocatalytic reactions.

### 3.4. Photocatalytic Performance Analysis

**Figure 7:**
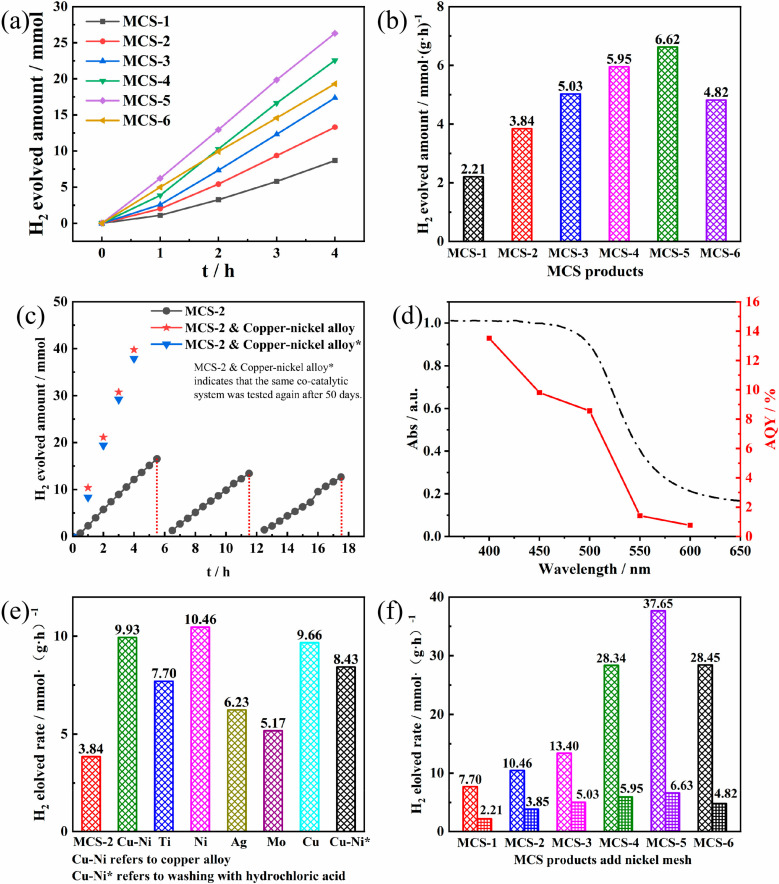
(**a**,**b**) H_2_ production activity of MCS products with different TAA addition amounts. (**c**) Photostability study and (**d**) apparent quantum yield for H_2_ production for MCS-2 (5 mg) from 50 mL of Na_2_SO_3_ (0.25 M)-Na_2_S (0.35 M) aqueous suspension system. (**e**) H_2_ production activity of MCS-2 with different metal mesh contents. (**f**) Comparison diagram of hydrogen production before and after the addition of the nickel mesh.

In this work, MCS-2 was selected as a reference for H_2_ production due to its excellent stability and reproducibility of the results. Meanwhile, under visible light (λ ≥ 420 nm) irradiation, the photoreaction conditions for the generation of H_2_ production were optimized using a mixture of sodium sulphide Na_2_S (0.35 M) and sodium Na_2_SO_3_ (0.25 M) solution [32]. After optimizing, the MCS exhibited rather excellent hydrogen production performance under visible light irradiation, as shown in Figure 7a,b. It can be seen that the hydrogen production activity of the MCS products increased with the TAA values from 4 mmol to 15 mmol and then decreased slightly once the TAA reached 20 mmol. This result is in agreement with the UV-vis diffuse reflection absorption spectrum. Thus, the variation in hydrogen producing activity of the MCS products may be due to the differences in TAA addition, resulting in changes in the morphology and composition of the samples. For example, it was discussed in the XRD section that the addition of different amounts of TAA would have an effect on the yields of MCS solid solution. This, in turn, has an effect on the absorption of light and charge generation/separation processes of the sample.

Figure 7c shows the photostability of the present photoreaction system. The average H_2_ production activity of MCS-2 in the first cycle of 6 h process was 3.13 mmol·(g·h)^−1^ and the average H_2_ production activity remained at 77.80% after the third cycle. In addition, the highest apparent quantum yield (AQY) of 13.5% was achieved by the MCS-2 sample under 400 nm monochromatic light irradiation, as can be seen in Figure 7d. Among the reported sulphide-based photocatalysts, this quantum efficiency value is relatively high. The AQY value tends to decrease with the enhancement of monochromatic light wavelength, which is consistent with the trend of the UV-vis spectrum. The AQY values of MCS-2 under 450 nm, 500 nm, 550 nm, and 600 nm irradiation were calculated to be 9.8%, 8.6%, 1.4%, and 0.8%, respectively. The high photocatalytic H_2_ production efficiency of MCS samples can not only be attributed to its good light absorption ability. The high AQY values can be ascribed to the components’ synergistic effect, such as the excellent charge separation efficiency on the interfaces of solid solution, enabling the rapid transfer of photogenerated electrons from MCS samples under light irradiation and thus improving the efficiency of hydrogen production.

Figure 7e exhibits the effect on the photocatalytic activity of MCS-2 after the addition of the metal mesh. It can be seen that the addition of various metal meshes leads to an increase in hydrogen production. Among the meshes, the high-purity nickel mesh has the greatest improvement in hydrogen production, reaching 2.72 times that of the original. Meanwhile, the improvement obtained from the high-purity metallic molybdenum mesh is the smallest, only 1.34 times that of the original. For the other meshes, such as Ti mesh, the high-purity Ag mesh and the high-purity Cu mesh, their improvements are 2.00 times, 1.62 times, and 2.51 times higher than that of the original. It is worth noting that the sacrificial agents used in the hydrogen production test contains a large amount of inner S^2−^, which will react to produce black Ag_2_S when placed into the silver mesh, resulting in changes to the physicochemical properties of the silver mesh. Therefore, this result is only for reference. In order to accurately measure the promotion effect of the silver mesh on the catalytic reaction, it needs to be measured in another appropriate system. In addition, for the high-purity nickel mesh and high-purity copper mesh with the largest proportion of hydrogen production increase, when they were formed into a copper–nickel alloy with an atomic percentage of 66.86:33.14 and put into the system, the increase in hydrogen production was in between the two constituent high-purity metal meshes, and hydrogen production decreased after the copper–nickel alloy was washed with sufficient hydrochloric acid. Additionally, we investigated the stability of the co-catalytic system, and the results are shown in Figure 7c. After the hydrogen production test was performed for MCS-2 and the copper–nickel alloy mesh, the system was kept in the shade for 50 days, then the H_2_ production test was conducted again. The results showed that the hydrogen production activity was still maintained at 96.72%, indicating that the co-catalytic system has high value for practical industrial applications.

Because the catalytic system formed by high-purity metallic nickel mesh/MCS has the highest increase in hydrogen production, we selected the high-purity metallic nickel mesh and put it into MCS catalytic system under different amounts of TAA addition. In Figure 7f, the results show that the maximum hydrogen production still corresponds that of MCS-5, which is 37.65 mmol·(g·h)^−1^. When the high-purity Ni metal mesh was placed in each catalytic system, the increase in hydrogen production of each reaction system was not a fixed value but fluctuated between 2.66 and 5.90 (an average increase of 4.20 times). This indicates that the Fermi energy level of the metal is not the only influencing factor in the metal mesh–MCS catalytic system. The characteristics of the material itself, such as the number of active sites and the light absorption capacity in the visible light range will also affect the hydrogen production efficiency of the whole system.

### 3.5. Photoelectrochemical Measurements

**Figure 8:**
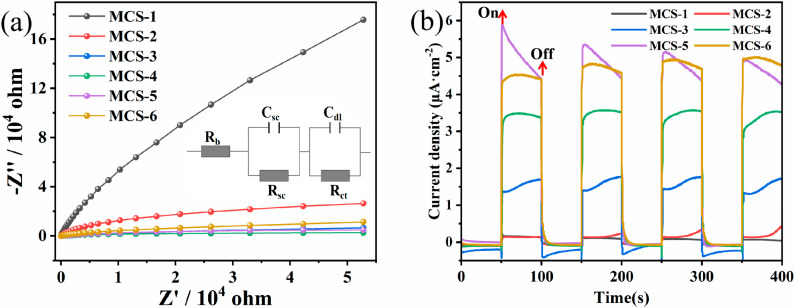
(**a**) Electrochemical impedance spectroscopy (EIS) and the equivalent circuit of MCS. (**b**) The periodic on/off photocurrent responses of MCS.

Electrochemical impedance spectroscopy (EIS) was used to provide more evidence for charge transfer efficiency of catalysts. Figure 8a shows a Nyquist plot conforming to the equivalent Randle circuit, and the fitted values of R_b_, C_sc_, R_sc_, C_dl_, and R_ct_ are listed in Table 4. Usually, the small radius curves indicate a higher charge transfer efficiency of the samples. It can be seen that MCS-4, MCS-5, and MCS-6 have smaller radius curves when compared to MCS-1 and MCS-2. Therefore, they have lower resistance and can transfer charge quickly to improve H_2_ production activity.

In Table 4, the resistance of the whole reaction system is expressed by R_b_, C_sc_ is the capacitance of the interface layer formed on the electrode surface due to charging, R_sc_ is the resistance caused by charging, C_dl_ is the electric double-layer capacitance caused by the change of ion concentration, and R_ct_ is the resistance during the transfer process. The above analytical results remain consistent with those of the UV-vis and photocatalytic performance.

The electron–hole separation of different MCS materials was investigated by a series of photochemical measurements, and Figure 8b shows the photocurrent response intensity of the samples. The photocurrent in this test was generated by the migration of photocarriers to the catalyst surface, and the vacancies were consumed by the sacrificial agent. The weak photocurrents of MCS-1 and MCS-2 under visible light irradiation indicate their low photogenerated carrier production efficiency, which can be attributed to their weak visible light absorption. Similar to the above results, the optical current density of MCS-5 is also the highest among all samples. This is due to the fact that MCS-5 has the smallest R_ct_ in all samples, so its optical carrier migration rate is the highest. In order to show the difference of photocurrent of various products more intuitively, the approximate change rate of current density was obtained by subtracting the average dark current from the average of the optical current. The results are listed in Table 5.

### 3.6. Energy Band Structure and Photocatalytic Mechanism

The value of the flat-band potential E_fb_ of the MCS solid solution was determined using the Mott–Schottky relationship (Equation (4)) [32]. The energy band structure of the MCS solid solution and the mechanism of action of the catalytic system were investigated on this basis. In particular, the space charge layer capacitance has been represented as:

(4) $$C^{-2}=(2/e\varepsilon \varepsilon_{0}N_{d}{)[V}_{a}{-E}_{\mathrm{fb}}-\mathrm{kT}/e]$$

where e is the electronic charge, ε is the dielectric constant, ε_0_ is the vacuum tolerance, N_d_ is the electron donor density, V_a_ is the applied potential, and E_fb_ is the flat-band potential [12].

**Figure 9:**
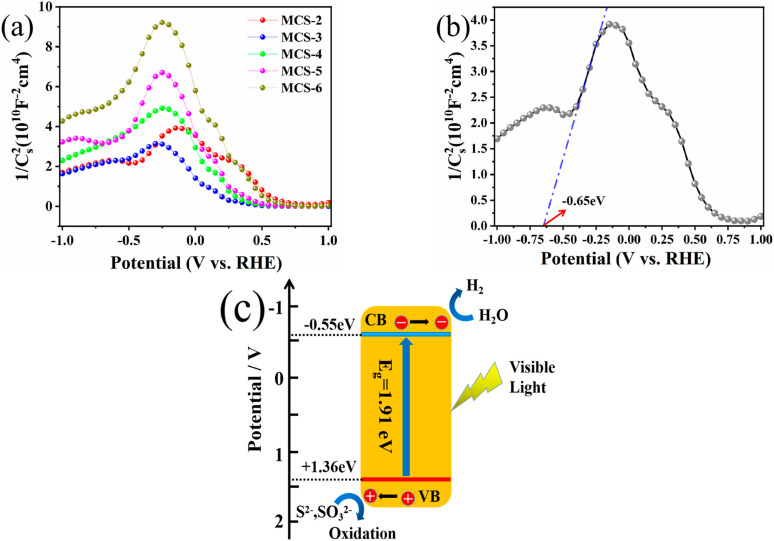
(**a**) Mott–Schottky curves of different MCS products. (**b**) Mott–Schottky curves of MCS-2. (**c**) Energy band diagram of MCS-2 alone.

As can be seen in Figure 9a, there is a change in the slope of the plotted curve from positive to negative, which is due to the formation of p–n junctions [25]. Besides, the X-axis intercepts of the Mott–Schottky plots of the different MCS samples are different. Among them, the E_fb_ value of MCS-2 is −0.65 eV vs. Ag/AgCl (E^θ^ = 0.205 eV vs. NHE, saturated KCl solution) in Figure 9b [35,36], so its E_fb_ value can be calculated to be −0.65 + 0.205 = −0.45 eV vs. NHE. Since the bottom of the conduction band (CB) is more negative (−0.1 V) than E_fb_ for certain n-type semiconductors [37,38], the CB level (E_CB_) of MCS can be estimated to be −0.55 eV [36]. According to the previous conclusions, the absorption edge of MCS-2 lies near 600 nm, and the exact band gap value corresponds to 1.91 eV (Figure 6a,b). As such, its valence band (VB) level (E_VB_) can be calculated to be +1.36 eV according to the formula (E_VB_ = E_g_ + E_CB_) [35,36,39]. To summarize, the potential energy diagrams for MCS-2 solid solution are shown in Figure 9c.

**Figure 10:**
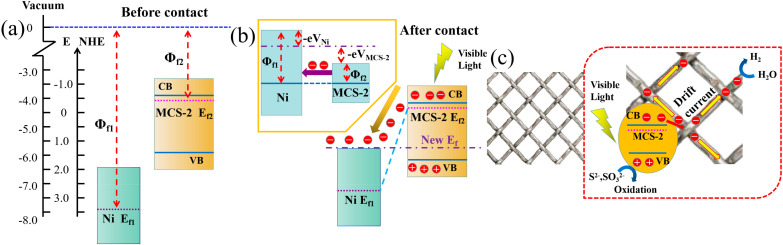
(**a**) The respective energy band structures of Ni and MCS-2 before contact are presented. (**b**) The Fermi energy level equilibrium process of Ni and MCS-2 after contact is proposed. (**c**) The photocatalytic mechanism of Ni/MCS-2 composites under visible light irradiation.

Based on the various characterization and performance results of MCS and metal mesh mentioned above, the photocatalytic mechanism of the whole system was discussed on the basis of the energy band structure. Taking high-purity metal nickel-loaded MCS-2 as an example (Figure 10a), the Fermi level of MCS-2 (−0.45 V vs. NHE) calculated by formula E(eV) = −4.5 − E(V) is higher than that of Ni (−7.98 eV vs. vacuum, obtained from the CASTEP calculation). Thus, when they come in contact with each other, electrons will migrate from MCS-2 to nickel to reach a new Fermi level equilibrium (Figure 10b). When MCS-2 was irradiated by visible light, photoexcited electrons would migrate from the VB of MCS-2 to nickel, while leaving the equal amount of holes in VB. Meanwhile, according to the Fermi–Dirac distribution function, the new equilibrium Fermi energy level of metal nickel/MCS-2 must be lower than the CB of MCS-2, so the photoexcited electrons can be transferred from CB of MCS-2 to nickel. The photoexcited electrons transferred to the high-purity metal nickel mesh directly form a drift current, which concentrates the nickel mesh with electrons and the MCS-2 with holes. Therefore, metal nickel acts as an electron trap to effectively prevent the recombination of the carriers [40,41]. These photoexcited electrons transferred to the nickel surfaces can eventually be trapped by the adsorbed H^+^ and form H_2_.

Based on the above analysis, the flow of electrons from MCS-2 to nickel will produce additional electrostatic potential energies (−eV_Ni_ > 0 and −eV_MCS-2_ < 0) and, finally, bring the Fermi level close, and even reach agreement (Figure 9b). Therefore, the different Fermi levels with metals and semiconductors can determine the level of electrostatic potential energy and affect the flow of electrons. This was also proven in H_2_ production. For example, the Fermi level of copper (−5.82 eV vs. vacuum, obtained via the CASTEP calculation) is higher than that of nickel (−7.98 eV vs. vacuum), which leads to the lower Fermi level difference between copper and MCS-2 than that of nickel. This weakens the flow of electrons and reduces H_2_ production. The new Fermi level formed by copper nickel alloy, according to the above theory, is just between the two original Fermi levels, similar to how the H_2_ production of the copper–nickel alloy is also between the H_2_ production with the addition of the nickel mesh and copper mesh. After the copper–nickel alloy is washed with sufficient hydrochloric acid for a sufficient time, because the concentration of hydrochloric acid is not enough to make copper form the H_2_CuCl_4_ complex, only nickel is washed away in large quantities. This increases the Fermi level of the alloy and reduces H_2_ production.

**Figure 11:**
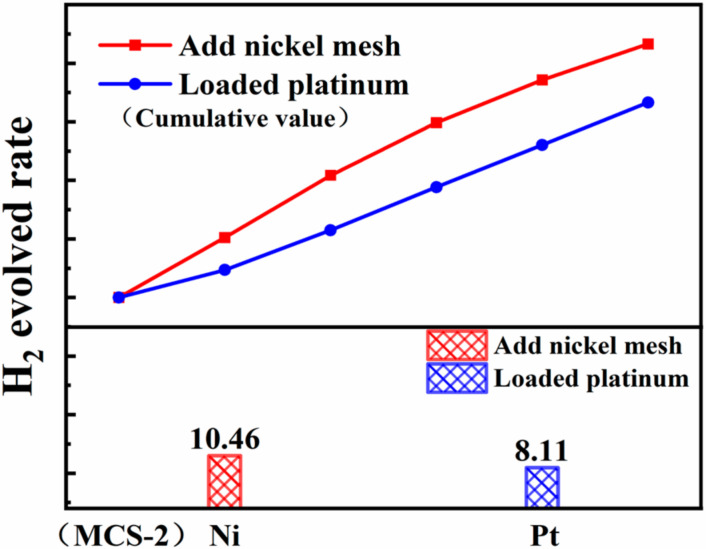
Comparison diagram of hydrogen production through addition of the nickel mesh and loaded platinum.

Several studies [42,43] have pointed out that by synthesizing small-sized metal particles as co-catalysts, the interfacial charge transfer distance can be shortened and the probability of carrier recombination can be reduced, thus effectively increasing the rate of photocatalytic hydrogen production. In order to compare the difference of catalyst promotion between the small-size metal co-catalyst and the metal mesh, we added 0.005 g/mL H_2_PtCl_6_ solution (50 μL) to the MCS-2 catalytic system and used a 300 W xenon lamp illuminated for 10 min to successfully load small-size metal platinum on MCS-2. The results in Figure 11 show that the group with nickel mesh has a higher improvement in the hydrogen production performance of MCS-2. It is speculated that the large-size metal mesh causes the diffusion range of drift current to become larger and the number of reduction sites of hydrogen to increase.

In conclusion, the addition of the metal nickel mesh to the catalytic system can improve the hydrogen production performance of the system better than loading small-size metal platinum cocatalyst. Due to the relatively low price of metal mesh, its application in industry is more promising.

## 4. Conclusions

In summary, novel MCS products were fabricated using a simple one-pot solvothermal process. The material was further characterized using XRD, HRTEM, etc. The results showed that the products are an MCS solid solution rather than a simple MnS/CdS mixture and by enhancing the TAA value from 4 mmol to 15 mmol, the MCS products show increasing H_2_ production activity, up to 6.63 mmol·(g·h)^−1^. By adding different kinds of metal mesh, the catalytic performance of the system can be greatly improved. Among them, it can be observed that the improvement of the Ni mesh is the best, up to 2.72 times greater. When Ni mesh was added to different MCS catalytic systems, the average hydrogen production increased 4.20 times, and the maximum hydrogen production was 37.65 mmol·(g·h)^−1^. In addition, the Ni(mesh)/MCS-3 catalytic system significantly improved the production performance of H_2_ and surpassed MCS-3 loaded with small-scale metal Pt. After a full discussion, it can be considered that the photoexcited electrons transferred to the high-purity metallic nickel mesh directly form a drift current, which causes the Ni mesh to enrich electrons and MCS to enrich holes. As such, Ni acts as an electron trap to suppress the recombination of electrons and holes and, finally, effectively prevents the recombination of carriers. This study not only proposed a novel catalytic system consisting of an MCS solid solution and metal mesh for the first time, but also shown that low-cost metal mesh can have similar or better performances than small-particle-size noble metal as an efficient co-catalyst for photocatalytic hydrogen production.

## Figures and Tables

**Table 1:** Assignment of catalyst names.

Samples	Thioacetamide (mmol)
MCS-1	4
MCS-2	6
MCS-3	8
MCS-4	10
MCS-5	15
MCS-6	20

**Table 2:** All the element contents of the copper nickel alloy.

Element	Line Type	Weight %	Atomic %
Ni	L	65.09	66.86
Cu	L	34.19	33.14

**Table 3:** Visible light absorption performance (600–800 nm).

Samples	Absorbance (a.u.)
MCS-1	0.133
MCS-2	0.166
MCS-3	0.297
MCS-4	0.316
MCS-5	0.373
MCS-6	0.304

**Table 4:** Equivalent circuit parameters of MCS.

Different Samples	R_b_ (ohm/cm^2^)	C_sc_ (F/cm^2^)	R_sc_ (ohm/cm^2^)	C_dl_ (F/cm^2^)	R_ct_ (ohm/cm^2^)
MCS-1	5.911	1.47 × 10^−4^	801.5	7.05 × 10^−5^	4.89 × 10^5^
MCS-2	36.46	2.47 × 10^−4^	982.6	2.52 × 10^−4^	4.36 × 10^4^
MCS-3	39.82	2.22 × 10^−3^	249.3	1.45 × 10^−3^	9321
MCS-4	41.02	2.36 × 10^−3^	265.4	2.42 × 10^−3^	4765
MCS-5	31.69	1.79 × 10^−3^	1.56 × 10^4^	2.93 × 10^−3^	185.3
MCS-6	40.35	7.28 × 10^−4^	566.7	8.38 × 10^−4^	1.87 × 10^4^

**Table 5:** The approximate change rate of current density.

Samples	Dark Current	Optical Current	Current Density
MCS-1	−2.80 × 10^−7^	−1.01 × 10^−7^	1.79 × 10^−7^
MCS-2	−2.27 × 10^−7^	2.73 × 10^−7^	3.63 × 10^−7^
MCS-3	−1.07 × 10^−7^	1.52 × 10^−6^	1.74 × 10^−6^
MCS-4	−5.24 × 10^−8^	3.41 × 10^−6^	3.46 × 10^−6^
MCS-5	−9.06 × 10^−8^	4.74 × 10^−6^	4.84 × 10^−6^
MCS-6	−1.71 × 10^−8^	4.73 × 10^−6^	4.74 × 10^−6^
